# Application of Direct Agglutination Test (DAT) and Fast Agglutination Screening Test (FAST) for sero-diagnosis of visceral leishmaniasis in endemic area of Minas Gerais, Brazil

**DOI:** 10.1186/1475-9292-4-4

**Published:** 2005-06-14

**Authors:** Eduardo S Silva, Gerard J Schoone, Celia MF Gontijo, Reginaldo P Brazil, Raquel S Pacheco, Henk DFH Schallig

**Affiliations:** 1 Universidade do Estado de Minas Gerais, Fundação Educacional de Divinópolis, Divinópolis, MG, Brasil; 2 KIT (Koninklijk Instituut voor de Tropen / Royal Tropical Institute) KIT Biomedical Research, Meibergdreef 39, 1105 AZ Amsterdam, The Netherlands; 3 Centro de Pesquisas René Rachou-Fiocruz, Belo Horizonte, MG, Brasil; 4 Departamento de Bioquímica e Biologia Molecular, IOC/ Fiocruz, Rio de Janeiro, RJ, Brasil

## Abstract

**Background:**

The direct agglutination test (DAT) has proved to be a very important sero-diagnostic tool combining high levels of intrinsic validity and ease of performance. Otherwise, fast agglutination screening test (FAST) utilises only one serum dilution making the test very suitable for the screening of large populations.

**Results:**

We have tested FAST and DAT for the detection anti-*Leishmania *antibodies in serum samples from patients with American visceral (AVL) and cutaneous leishmaniases (ACL) in Minas Gerais State, Brazil. The DAT on serum and blood samples of confirmed AVL patients found all samples positive at a serum dilution of ≥ 1:800. This dilution was subsequently used as cut off value in the study. The blood and serum samples of these confirmed patients could also be clearly read in FAST using a 1:100 dilution with the same high sensitivity. DAT and FAST were not able to detect significant amounts of antibodies in samples from ACL patients and are not suitable for the diagnosis of this manifestation of the disease.

**Conclusion:**

We suggest that both DAT and FAST are very practical diagnostic tools for the sero-diagnosis of AVL under rural conditions as both serological tests do not require sophisticated equipment, a cold chain and are very simple to perform.

## Background

American visceral leishmaniasis (AVL) a protozoan disease caused by *Leishmania chagasi *parasites, constitutes a major health problem in Brazil. In the last few years the number of human cases of AVL in the metropolitan region of Belo Horizonte (MRBH), state of Minas Gerais, Brazil has increased, indicating an elevation in the transmission rate of the disease [1]. Dogs and fox are considered to be the main reservoirs host for this parasite [2,3]. The diagnosis of AVL is based on clinical-epidemiological characteristics, by microscopical demonstration of the parasite in biopsies of aspirates, indirectly by serological tests and culturing or molecular methods like the polymerase chain reaction (PCR) [4-6]. Several techniques can be used for the sero-diagnosis of AVL. The indirect fluorescence technique (IFAT) was the technique of choice until 1974 [7,8]. Since then counter-current immuno-electrophoresis and enzyme-linked immunosorbent assay (ELISA) have been found to be powerful tools for the sero-diagnosis of leishmaniasis [9]. In addition, several other serological tests have been developed. The direct agglutination test (DAT) has proved to be a very important sero-diagnostic tool combining high levels of intrinsic validity and ease of performance [10-12]. The test uses whole, stained promastigotes either as a suspension or in a freeze-dried form [10,11,13,14]. By using the freeze-dried antigen, logistic problems, such as the need of a cold chain for storage of antigen, are avoided, making the DAT very suitable for use under field conditions.

Although the direct agglutination test (DAT) for the sero-diagnosis of visceral leishmaniasis has a high sensitivity and specificity [6,13,14], it still has some limitations like the relative long incubation time (18 h) and the need for serial dilutions of blood or serum. In order to circumvent these problems Schoone *et al. *[15] developed a fast agglutination-screening test (FAST) for the rapid detection of anti-*Leishmania *antibodies in serum samples and in blood collected on filter paper. The FAST utilises only one serum dilution (qualitative result) and requires 3 hours of incubation. This makes the test very suitable for the screening of large populations.

The increasing importance of AVL in man and its high rates of lethality in the Metropolitan Region of Belo Horizonte (MRBH) indicate that a rapid and relatively simple method is needed for the routine diagnosis of this disease. The objective of this study was to evaluate DAT and FAST as potential AVL diagnostic methods using clinical samples from this region in Brazil.

## Materials and methods

### Serum samples

This study was carried out utilising serum samples from different patients groups from metropolitan region of Belo Horizonte (MRBH), Minas Gerais State and some control samples from other regions (see below). Samples and information of patients (age, sex, symptomatology, clinical, address) were sent to the laboratory through the existing health care system of MRBH, Minas Gerais, Brazil. Written consent was obtained from the patient or their relative for this study.

The following groups of samples were included in the present study:

1. Serum samples of AVL patients parasitologically confirmed (microscopical examination from bone marrow aspirate smears) (n = 16).

2. Serum samples of patients clinically suspected of AVL (fever, spleen enlargement, pallor, weight loss), but not parasitologically confirmed (n = 99).

3. Serum samples of patients presenting with active cutaneous leishmaniasis lesions (n = 85).

4. Serum samples of patients with confirmed Chagas' disease (n = 12)

5. Serum samples of patients (Uganda) with confirmed African trypanosomiasis (n = 5)

6. Serum samples of patients (Kenya) with confirmed *P. falciparum *malaria (n = 5)

7. Serum samples of confirmed toxoplasmosis patients (The Netherlands) (n = 5)

8. Serum samples of apparently healthy individuals from an AVL endemic region in Brazil (n = 19)

9. Serum samples of healthy blood donors from a non-endemic region (The Netherlands; n = 5)

### Serology

The presence of *Leishmania *antibodies in all the serum samples was determined by FAST and DAT. A sub-set of the samples (groups 1 – 3) were also analysed with IFAT. Antigen for FAST and DAT were prepared as described earlier [13,15]. The FAST was performed according to the protocol described previously [15]. In brief, serum samples were diluted 1:100 in physiological saline (NaCl 0.85%) to which 0.78% β – mercaptoethanol was added in a V-shaped microtitre plate (Greiner, Germany). Next, 20 μl of this 1:100 dilution was transferred to another well of the V-shaped microtitre plate and 20 μl FAST antigen (2 × 10^8 ^promastigotes/ml) was added. The plate was carefully shaken, covered with a lid and allowed to incubate for 3 hours at room temperature after which the results were read. Appropriate positive and negative controls were always included on each plate.

The DAT was performed essentially as previously described by [10,13]. In brief, the samples were diluted in physiological saline (0.9% NaCl) containing 0.78% β – mercaptoethanol. Two-fold dilution series of the sera were made in a V-shaped microtitre plate, starting at a dilution of 1:100 (step 1) and going up to a maximum serum dilution of 1:102.400 (step 11). Well 12 was used as a negative control. Fifty μl DAT antigen (concentration of 5 × 10^7 ^parasites per ml) was added to each well containing 50 μl diluted serum and the results were read after 18 hours of incubation. The cut-off value of the DAT was set at >1:800.

The IFAT was, due to logistic problems, only performed on a selection of the serum samples using a commercial kit for the diagnosis of human leishmaniasis (Fiocruz/Bio-Manguinhos, Rio de Janeiro, Brazil). IFAT was performed according to the instructions of the manufacturer for detection of antibodies in serum diluted from 1:40 up to 1:640.

### Statistical analysis

The sensitivity and specificity of the DAT and FAST in the present study were calculated as follows: Sensitivity = TP/(TP+FN) × 100% and Specificity = TN/(TN+FP) × 100%. Where TN represents true negative, TP true positive, FN false negative and FP false positive. The sensitivity of the two tests, FAST and DAT, was assessed with sera from confirmed AVL patients (n = 16). Sera of healthy controls (n = 24) and sera of patients with confirmed other diseases (n = 112) were used to determine the specificity of DAT and FAST.

The degree of agreement between FAST, DAT and IFAT was determined by calculating Kappa (κ) values with 95% confidence intervals using Epi-info version 6. Kappa values express the agreement beyond change and a κ value of 0.21 – 0.60 represents a fair to moderate agreement, a κ value of 0.60 – 0.80 represents a substantial agreement and a κ>0.80 represents almost perfect agreement beyond change [16]. The calculation of the degree of agreement between DAT and FAST was based on all serum samples, whereas the κ values for DAT -IFAT and FAST – IFAT were only based on the results obtained with the confirmed and suspected AVL serum samples.

## Results

The results of the serological analysis with DAT and FAST are presented in Tables 1 and 2, respectively. The sensitivity of the DAT in the present study was calculated to be 100% and its specificity 97.8% (3 false positive results). The FAST had a sensitivity of 100% and a specificity of 92.5% (11 false positive results). The results of the IFAT testing are presented in Table 3. The sensitivity and specificity of the IFAT could not be adequately calculated as a sufficient number of negative samples was not analysed with this test. It should be noted that IFAT tested 1 confirmed AVL patient negative and 7 patients only had a low IFAT titre. In contrast, IFAT found about 50% of all confirmed ACL patients sero-positive, whereas neither DAT nor FAST was able to detect antibodies in these serum samples. The DAT found 77 out of 99 samples of patients suspected (but not parasitologically confirmed) of AVL positive. It was observed that 79/99 patients were FAST positive and 84/99 patients were IFAT positive with serum dilutions varying from 1:40 to 1:640.

**Table 1:** Direct agglutination test (DAT) results for anti-*Leishmania *antibodies in various patients groups.

	Serial dilution series								
Patient group	≤ 800	1600	3200	6400	12800	25600	51200	102.400	Total (n)

1 confirmed AVL	-	-	1	-	4	1	3	7	16
2 suspected AVL	22	-	3	9	8	15	5	37	99
3 confirmed CL	84	-	1	-	-	-	-	-	85
4 confirmed Chagas' disease	10	2	-	-	-	-	-	-	12
5 confirmed trypanosomiasis	5	-	-	-	-	-	-	-	5
6 confirmed malaria	5	-	-	-	-	-	-	-	5
7 confirmed toxoplasmosis	5	-	-	-	-	-	-	-	5
8 healthy endemic controls	19	-	-	-	-	-	-	-	19
9 healthy non-endemic controls	5	-	-	-	-	-	-	-	5

**Table 2:** Fast agglutination screening test (FAST) results for anti-*Leishmania *antibodies in various patients groups.

Patient group	FAST negative	Fast Positive	Total (n)
1 – Confirmed AVL	0	16	16
2 – Suspected AVL	20	79	99
3 – Confirmed CL	82	3	85
4 – Confirmed Chagas' disease	6	6	12
5 – Confirmed trypanosomiasis	4	1	5
6 – Confirmed malaria	5	0	5
7 – Confirmed toxoplasmosis	5	0	5
8 – Healthy endemic controls	18	1	19
9 – Healthy non-endemic controls	5	0	5

**Table 3:** Indirect Immunofluoresence Test (IFAT) results for anti-*Leishmania *antibodies in serum of sub-set of the different patients groups (NR = no reaction).

Patient group	NR	1:40	1:80	1:160	1:320	1:640	Positive/Total (n)
1 – Confirmed AVL	1	1	6	-	6	2	15/16
2 – Suspected AVL	15	7	20	20	23	14	84/99
3 – Confirmed ACL	37	3	23	12	9	1	48/85

A high degree of agreement (96%) was observed between FAST and DAT (Table 4a). The agreement beyond change (κ value) was 0.92. In addition, substantial agreement was observed between DAT and IFAT (93%; Table Table 4b) or FAST and IFAT (94%; Table Table 4c), with κ values of 0.75 and 0.80, respectively.

**Table 4:** Comparison between DAT, FAST and IFAT using serum samples from suspected visceral leishmaniasis patients from the Belo Horizonte Metropolitan Region.

A. Comparison between DAT and FAST			
	FAST +	FAST -	Total
DAT +	96	0	96
DAT -	10	145	155
Total	106	145	251
Comparison between DAT and IFAT			
	IFAT +	IFAT -	Total
DAT +	92	1	93
DAT -	7	15	22
Total	99	16	115
C. Comparison between FAST and IFAT			
	IFAT +	IFAT -	Total
FAST +	94	1	95
FAST -	5	15	20
Total	99	16	115

## Discussion

In view of the public health importance of AVL and the inherent difficulties of conventional diagnosis techniques, we evaluated in the present study the performance of the sero-diagnostic tests, DAT and FAST. Both test displayed a very high sensitivity and specificity corroborating with previous studies [10,13-15]. It is noted that the sensitivity of DAT and FAST observed in the present study was determined using a relatively low number of confirmed patients, and therefore 100% sensitivity is not claimed.

The antigen on which DAT and FAST are based is a strain of *L. donovani*, whereas human and canine visceral leishmaniasis in Brazil is caused by *Leishmania chagasi*, both species belonging to the *L. donovani *complex. Apparently the use of an heterologous antigen did not affect the performance of both tests for the detection of anti-*Leishmania *antibodies in Brazilian AVL patients. The DAT found all parasitologically confirmed AVL patients positive at a serum dilution of ≥ 1:800, which was subsequently used as the cut off dilution in the present study. This serum dilution is comparable to the cut off dilutions found in several other studies [13,14]. The FAST also found all confirmed cases positive, which should be the case as this test is intended as a screening test that should not miss any AVL patient. This result is even better than a previous evaluation of the FAST in which some cases were missed [15].

We have also compared the performance of IFAT with DAT and FAST on serum samples of confirmed AVL cases and suspects. The IFAT missed one confirmed patient that had a very high DAT serum dilution. On the other hand, IFAT found slightly more suspected ALV patients positive (84/99) than DAT (77/99) or FAST (79/99) did. However, there was in general a very good agreement between the performance of the three tests with regard to the seo-diagnosis of AVL. In contrast, both FAST and DAT found only a very limited number of ACL cases sero-positive. IFAT found approximately 50% of these cases positive, albeit with generally very low serum titres. DAT and FAST based on *L. donovani *antigen are not suitable for the sero-diagnosis of ACL [13].

## Conclusion

As final remarks, we can conclude that DAT and FAST are very suitable tools for the sero-diagnosis of AVL. Both tests are easy to interpret, as well as being specific and sensitive. The DAT is very practical under field or rural conditions, as no specialised equipment is required nor a cold chain is necessary for the storage of antigen. In addition, the FAST requires only one serum dilution and the results can be read within 3 hours. The FAST can be used to screen large populations, for example in situations such as epidemics where large number of suspects are seen at the clinic or cases where immediate treatment is necessary.

## Competing interests

The author(s) declare that they have no competing interests.

## Authors' contributions

Silva ES performed the practical work as part of his PhD study, partly in Brazil and partly in the Netherlands, wrote the concept of the paper. Schoone GJ research technician performed part of the sample and data analysis, produced DAT and FAST antigen and invented the FAST test. Gonijo CMF took part in the study design, supervised practical work in Brazil and assisted in writing the concept of the paper. Pacheco RS and Brazil RP former supervisors of Silva ES took part in study design. Schallig HDFH supervised the practical work in the Netherlands, data analysis, final preparation of manuscript, corresponding author
